# The Importance of Mental Models in Implementation Science

**DOI:** 10.3389/fpubh.2021.680316

**Published:** 2021-07-06

**Authors:** Jodi Summers Holtrop, Laura D. Scherer, Daniel D. Matlock, Russell E. Glasgow, Lee A. Green

**Affiliations:** ^1^School of Medicine, University of Colorado, Aurora, CO, United States; ^2^Department of Family Medicine, University of Alberta, Edmonton, AB, Canada

**Keywords:** mental models, Implementation strategies, adaptation, assessment methods, context, barriers

## Abstract

Implementation science is concerned with the study of adoption, implementation and maintenance of evidence-based interventions and use of implementation strategies to facilitate translation into practice. Ways to conceptualize and overcome challenges to implementing evidence-based practice may enhance the field of implementation science. The concept of mental models may be one way to view such challenges and to guide selection, use, and adaptation of implementation strategies to deliver evidence-based interventions. A mental model is an interrelated set of beliefs that shape how a person forms expectations for the future and understands the way the world works. Mental models can shape how an individual thinks about or understands how something or someone does, can, or should function in the world. Mental models may be sparse or detailed, may be shared among actors in implementation or not, and may be substantially tacit, that is, of limited accessibility to introspection. Actors' mental models can determine what information they are willing to accept and what changes they are willing to consider. We review the concepts of mental models and illustrate how they pertain to implementation of an example intervention, shared decision making. We then describe and illustrate potential methods for eliciting and analyzing mental models. Understanding the mental models of various actors in implementation can provide crucial information for understanding, anticipating, and overcoming implementation challenges. Successful implementation often requires changing actors' mental models or the way in which interventions or implementation strategies are presented or implemented. Accurate elicitation and understanding can guide strategies for doing so.

## Introduction

*People's views of the world, of themselves, of their own capabilities, and of the tasks that they are asked to perform, or topics they are asked to learn, depend heavily on the conceptualizations that they bring to the task*.

*-Donald A. Norman (in Mental Models, p.7)*

Implementation science is concerned with adoption, implementation, and maintenance of evidence-based interventions and use of implementation strategies to facilitate translation into practice (1). The field has grown rapidly and has resulted in the development of frameworks, theories, and approaches that assist in overcoming challenges to implementation difficulties (2–4). One approach is to identify root causes of implementation challenges and then apply appropriate implementation strategies to overcoming these challenges (5). Another is to identify and study the mechanisms of action that explain how and why an implementation strategy works (6). Indeed, much recent work has involved the cataloging and selection of implementation strategies and identification of mechanisms or determents of implementation outcomes (6, 7). However, the field still struggles with ways to understand why interventions do and do not work (8). Certainly, frameworks, such as the Consolidated Framework for Implementation Research (CFIR) (5), iPARIHS (9), the Practical Robust Implementation and Sustainability Model (PRISM; an extension of RE-AIM) (10, 11), and the Exploration, Preparation, Implementation and Sustainability (EPIS) (12, 13) can point to places to look for challenges. These models and frameworks consider categories of where implementation challenges may be found, such as in the intervention itself, the internal setting and/or infrastructure, or the change strategies. Thus, these frameworks are good at telling us where to look, but not so good at helping us understand what we're seeing when we find it, or what to do about it. Therefore, there may be cross-cutting ways to view the *where* and the *why* of implementation challenges that examine more the “cognitive determinants” in addition to the settings, actors, intervention, and implementation strategy characteristics.

We propose considering the concept of mental models as one of those cross-cutting ways to view implementation challenges. A mental model is an interrelated set of beliefs that shapes a person's expectations for the future and how they understand the way the world works (14–18). Mental models shape how an individual thinks about or understands how something or someone does, can, or should function in the world (14). Thus, eliciting, understanding, and acting upon how different individuals and groups conceptualize mental models both individually and collectively can be critical in health care improvement because the mental models might reveal implementation challenges that seem unclear or intractable. We argue these mental models deserve attention, much like the attention that has been given to identifying challenges, such as having enough resources (money, time, people) to get an intervention to happen, or lack of knowledge or training. These challenges, though certainly important determinants (19), tend to have a more on-the-surface quality to them that make them easier to find and thus qualitatively different from characteristics that are perhaps more entrenched and more difficult to pinpoint, and perhaps also change, such as mental models.

In this paper, our purpose is to introduce dissemination and implementation (D & I) researchers to the general concept of mental models and encourage consideration in tackling implementation challenges. We propose that understanding mental models is both complementary to the above factors and may separately and uniquely advance the field of implementation science. We provide a brief description of what mental models are, methods for eliciting and measuring them, and speculate about potential strategies to influence, adapt, or at least understand them to enhance implementation efforts. To illustrate, we use the example of shared decision-making (20) as a multi-level complex intervention to describe and explain the concepts presented (21). We conclude by discussing directions for clinical application and future research.

## What Is a Mental Model?

The concept of a mental model is used somewhat differently across disciplines, and indeed there is a long and complex literature regarding the definition, presence of, understanding of, and use of mental models (22–25). An in-depth account of this vast literature is not possible within the space of this paper, but worth acknowledging that it exists. To that point, we provide a somewhat cursory definition so that we may focus on describing the ways in which mental models may influence implementation. We acknowledge that our conceptual definition of mental models is not an operational definition that could be used to select concrete forms of representation, elicitation, and analysis, but rather a starting point for understanding and entry into its use in implementation science. Therefore, we adopt the view that a mental model is a person's mental representation of the way some aspect of the world works (17, 25, 26). More specifically, mental models are comprised of interrelated memories, conceptual knowledge, and causal beliefs that create an understanding of how something works in the real world and forms expectations about future events. For example, many, if not most, individuals in the United States (US) have a mental model of a doctor's appointment that includes the steps of the encounter, what kinds of questions and examination to expect, how to interpret what the doctor says (and does not say), and how to interact with the doctor to obtain desired information, tests, and treatments. This particular set of conceptual knowledge, expectations, and causal beliefs is formed primarily by our personal experiences and might also be formed by the transfer of cultural knowledge through media and social networks.

Mental models can be held with varying degrees of specificity and stability and are built over time by our knowledge and experiences. For example, a physician is likely to have a very different mental model of an appointment than a person who has only been a patient. A person in a low-income country, or a person from a low-income background whose primary health care system contact has been with the emergency department, may have a different mental model of a health care appointment or may not have a mental model at all. As a result, they may have no expectations or different expectations for what will or should happen. To our knowledge, mental models have not been applied to patient-physician communication. It is possible that errors in communication might often be rooted in a lack of a shared mental model (as is well known in the organizational psychology literature) (27), in addition to (or instead of) more obvious problems, such as lack of a shared first language or other communication challenges (28). Also, even in dyad relationships, such as the patient and provider, there is often the influence of the mental models of many others that impinge on the mental models of the two in the room, such as caregivers, family members, office administrators, health system executives, community members and more.

Mental models are always inaccurate to some extent, insofar as they are heuristics and involve stereotypes and expectations to make sense of the world. They cannot encapsulate all aspects of the world and tend to make imperfect predictions. When commonly shared mental models make inaccurate predictions, it can be a source of scientific insight and psychological fascination. For example, a common incorrect mental model about the physical world is that heavier objects fall faster than lighter ones. This mental model results in the expectation that a 20-pound ball will fall faster than a one-ounce ball, an expectation that was disproved compellingly by Galileo, but that is so counterintuitive that we must continue to dispel it in schoolchildren today.

The beliefs that comprise mental models can be explicit or tacit. *Explicit* beliefs are sometimes called the “know-what” (29) because people know what they believe to be true and can claim it. *Tacit* beliefs, on the other hand, are beliefs that are difficult to articulate and can be hidden.

The beliefs and expectations that are formed by our mental models sometimes become obvious (i.e., less tacit) when they are violated. For example, a wait of 10–20 min to see a doctor might be within scope of expectations, but a 45-min wait would violate most US people's mental model about what is supposed to happen and would trigger a complaint or visit back to the check-in desk. In this example, a violation of a mental model results in behavior to identify how or why that violation occurred.

Mental models can be more or less complex, a feature that usually depends on the depth of a person's knowledge and experiences. They are comprised of beliefs that are “core” to the model vs. beliefs that are more peripheral and inessential. Mental models can be more or less accurate, and more or less adaptive, in the sense of forming accurate predictions about future events. A given mental model may be very adaptive and helpful in one context and maladaptive in another. They can be shared between many people, cultures, and subgroups as a result of common experiences; although, it is important to keep in mind that mental models that are widely shared are not necessarily accurate (harkening back to Galileo's experiment) (30).

Importantly, mental models have implications for how new information is accepted or rejected. For example, research has shown that diagnostic labels can trigger patients to apply broad mental models which result in expectations for both treatment and disease progression (31). This creates a communication problem when the recommended course of action is one that the patient's mental model predicts will result in a bad outcome. For example, a recommendation of active surveillance for “stage 0 breast cancer” will seem safe and prudent under a physician's mental model but dangerous and frightening under a patient's mental model of what cancer is and how it progresses (32), leading to a communication breakdown. Patients must choose to either change the mental model to incorporate the new information or change their view of the information to fit with the existing mental model, the latter of which could include rejecting, ignoring, or reinterpreting the new information (33). When new information contradicts a core belief of the mental model, it is usually far easier to reject the new information. Hence, mental models have important implications for learning and for disbelief and resistance to and/or misunderstanding new information.

## How Mental Models Create a Barrier to Implementation: the Example of Shared Decision Making

Shared decision making is “a process of communication in which clinicians and patients work together to make informed healthcare decisions that align with what matters most to patients and their individual concerns, preferences, goals, and values” (34). Recently, there have been policy efforts and even payer mandates for shared decision making. However, despite strong data on the effectiveness of decision aids and shared decision making, the implementation of shared decision making in real world settings has been minimal, and when successful, is rarely sustainable. There has been a host of efforts to describe “barriers” to shared decision making (20, 35). Considering these barriers through the lens of mental models provides some clear examples of how the mental models of the parties involved in implementation could potentially be powerful drivers of implementation outcomes.

One example is the implantable cardioverter-defibrillator (ICD) for the prevention of sudden cardiac death for patients with heart failure. Recently, CMS mandated shared decision making with the use of a decision aid for this intervention (36). However, there has been resistance to shared decision making for implanted defibrillators by clinicians, and there are concerns that it is nothing more than a check box (37). In our ongoing work on implementation, our data suggest that a decision aid for shared decision making for ICDs does not fit well into physicians' mental models of heart failure management. For example, clinicians report things like (paraphrased from discussions to exemplify the underlying mental models), “I already do shared decision making”; “patients don't really have the capacity to understand the medical nuances”; or “I just don't have time for this.” Digging more deeply, we find that there are often unsaid (and powerful) mental models like, “my job is to help patients live as long as possible, and I want them to get the ICD” (38). In this case the physician's mental model includes a causal belief that the decision aid may turn patients away from the ICD, and shared decision making may conflict with the physician's model of their own role. At the patient level, we observe comments like: “I trust my doctor, and I'll do whatever she says”; or “This must be a good therapy because technologies are good” (39, 40), suggesting patients hold mental models that do not predict that shared decision making will bring them outcomes they value. The converse can also be true when someone has a mental model incorporating significant distrust, which could be one of the drivers of the observed disparities in ICD use.

**Figure 1:**
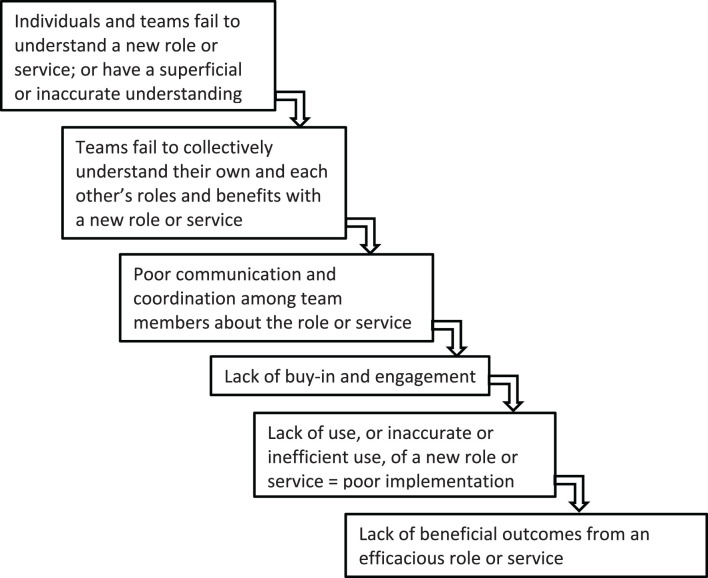
Potential pathway of failed implementation.

At the same time, mental models can be powerful drivers of successful implementation. In the case of shared decision-making for left ventricular assist devices (LVADs), we find evidence suggesting that physicians' mental models predict that LVADs can be both beneficial and harmful, that outcomes are better if patients are informed, and that the existing industry materials are biased and not a reliable source of information (41–45). The convergence of mental models in this space is serving as a powerful driver of adoption and implementation of shared decision making (46). The failure of such convergence can, conversely, also result in failed implementation. We present Figure 1 to demonstrate a common cascade of events stemming from lack of attention to mental model issues during the planning and implementation phases of a new intervention.

## How to Use Mental Models in Overcoming Implementation Challenges

If we accept that mental models can have a powerful influence over whether implementation happens or not, or happens well and is sustained, it is helpful to know how to identify an individual or group's mental model and assess if these mental models are facilitating or serving as barriers to implementation.

**Figure 2:**
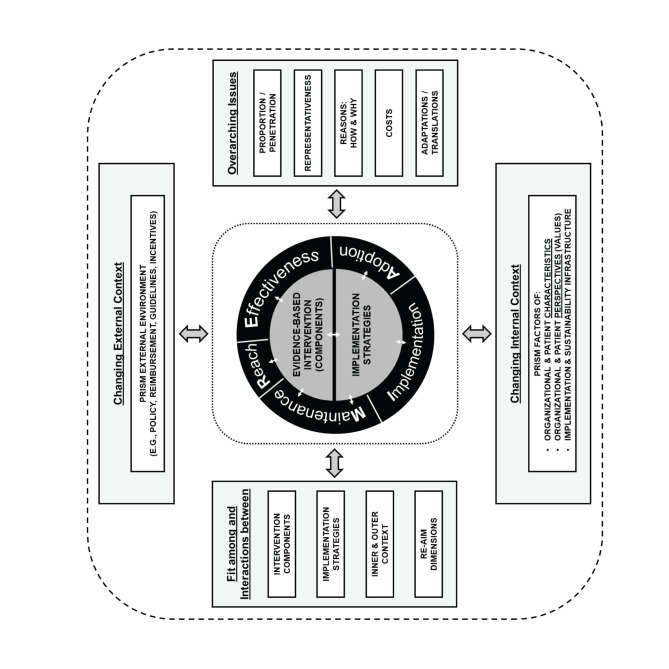
PRISM. Reprinted from Glasgow RE, Harden SM, Gaglio B, Rabin B, Smith ML, Porter GC, Ory MG, Estabrooks PA. RE-AIM Planning and Evaluation Framework: Adapting to New Science and Practice With a 20-Year Review. Front Public Health. 2019 Mar 29;7:64. doi: 10.3389/fpubh.2019.00064, an open-access article distributed under the terms of the Creative Commons Attribution License (CC BY).

To illustrate where mental model issues might be, we consider the use of an implementation science framework. The Practical, Robust, Implementation and Sustainability Model (PRISM; Figure 2) provides guidance regarding the contextual factors that influence the outcomes of reach, effectiveness, adoption, implementation and maintenance (RE-AIM outcomes, which are part of PRISM) (10). As noted in the introduction, mental models are cross-cutting and also multi-level. There may be mental model issues with the intervention itself at either the organization or recipient levels, or about the overall issue in question at the organization or recipient levels, with the implementation structure of the organization or community or the larger external environment. Perceptions about the changing external environment (e.g., expectations about what may be coming concerning guidelines or reimbursement for shared decision making) can increase or decrease use of an intervention. Finally, mental models of different amounts and types of resources needed for the PRISM category of Implementation and Sustainability Infrastructure (e.g., assigned responsibility, presence of audit and feedback systems) can lead to confusion or failure to provide sufficient support. For example, implementation of a decision aid to encourage shared decision making on a particular issue may be difficult in a practice where the majority of clinicians do not have a good mental model for how to use a decision aid, or if their mental model predicts that it will be disruptive in their practice, or if their mental model of the decision causes them to believe that the decision should not be shared. Therefore, it may be wise for the implementation researcher or practitioner to utilize an implementation science framework such as PRISM to identify possible places where mental model issues may be residing. This provides valuable information about what might be a possible avenue forward for resolving the issue. Some mental models are entrenched into such immutable values that changing them is unlikely or will take much effort over an extended time period.

Beyond where to look, how can you identify mental models? We provide Table 1 for suggestions for possible methods to elicit mental models. Some (such as interviews) are more helpful as a starting place when you don't know what the mental models might be; whereas, other methods (such as card sort) are more appropriate when some idea of the mental model might exist and what is needed is narrowing down to more explicit understanding. A key point is that the individual seeking to elicit the mental model tries to delve deep into understanding the root causes of the issue and tries to remain open to the nuanced information the participant is providing. Also, some methods have been developed specifically for eliciting mental models, such as cognitive task analysis; whereas, other more general methods, such as interviews, can sometimes lead researchers astray. It is much more likely in the latter case to get sincere and coherent narratives that just do not accurately capture important facets.

**Table 1:** Overview of common mental model elicitation methods.

**Method**	**Description**	**Considerations**
Survey	A series of questions with a closed ended response format (e.g., Likert scale from 1 to 5)	Efficient way to measure specific beliefs from a large group when one knows what the range of beliefs can be. Less suited for eliciting complex relationships in mental models. Models may have to be inferred.
Forced choice	A series of two-option choices presented to a participant, who is asked to choose one option over the other based on some criterion (e.g., preference, commonness, cost)	Suitable for eliciting rank and order among a narrower set of beliefs known beforehand. Less suited for eliciting complex relationships in mental models.
Card sort	A task in which the participant is given a set of cards with concepts to arrange in a way that is meaningful to him, either according to some set criterion (e.g., causal relationships) or not.	Suitable for eliciting grouping, sequencing, taxonomies, or processes. Cards can include images or be left blank for participants to fill in.
Semi-structured interviews	An interview in which a set of questions is prepared beforehand but can be deviated from opportunistically to learn more about the target topic.	More accurate and complete representations of mental models. Can capture complex relationships, but is time-consuming, expensive, and requires skilled interviewers.
Cognitive task analysis (47, 48)	A specific type of interview designed to elicit mental model and macrocognitive processes	Specifically designed to elicit and improve how teams function together in real world circumstances, but requires expert interviewers.
Causal mapping (49) and dynamic system diagraming (50, 51)	Mapping out causal relationships, feedback loops, and causal conditions/ or rules.	More accurate and complete representations of causal relationships in mental models, but is time-consuming, expensive, and requires skilled interviewers.
Delphi process	Multi-phase process of eliciting beliefs from several individuals, synthesizing responses, and sending the synthesis back for feedback.	Useful for building a shared mental model among non-co-located individuals and identifying points of disagreement. The process is slow and can be expensive (may need to pay experts).
Observation	Watching the performance of an individual or groups of individuals by an objective observer.	Because only behavior is observed and cognitions are not elicited, beliefs need to be inferred, unless recorded and combined with retrospective think aloud (see below).
Think aloud	A process in which the participants explains aloud what she is thinking as she performs a task (concurrent) or watches a recording of herself performing a task (retrospective).	Concurrent think aloud requires some practice by both the interviewer and participant and can sometimes interfere with the task. Elicits rich information about mental models in context.
Synthesizing documents	Using existing documents, such as reports of adverse events and near misses, to infer beliefs, and connections between beliefs.	Mental models are inferred and verification would require an additional method.

**Figure 3:**
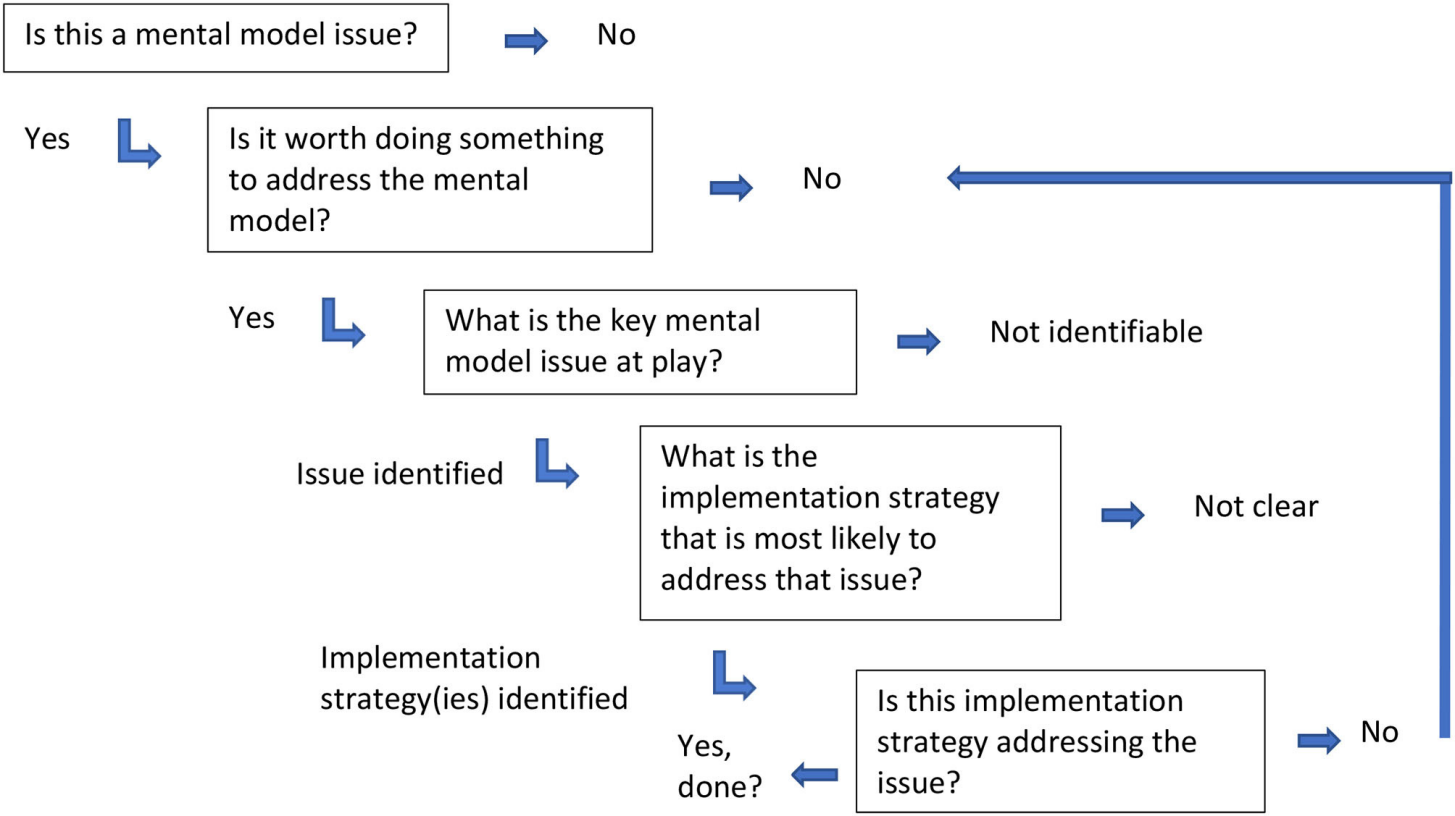
Mental model consideration pathway.

Once you have this information from the elicitation, then what do you do with it? How do you understand what you have identified? There are generally two steps left to complete your assessment: (1) analysis and (2) representation. Analysis can be undertaken in the way that most analysis is done, drawing upon quantitative and qualitative methods or a combination (52). For example, a quantitative approach might use a survey and compute descriptive statistics to describe the sample, or qualitative interviews might be analyzed using thematic analysis. These methods produce results about the mental model's information you have been examining. The key is an in-depth enough exploration of key individuals' mental models so that the implementation researcher can anticipate how these mental models might create barriers to implementation through using an “implementation pathway” as provided in Figure 3. Discovering if there is a mental model issue, if it is worth addressing, and what implementation strategy may affect the issue can be a useful place to start.

Representation is what you do with the information/results you have obtained from your elicitation and analysis methods. It is where you decide what to do. Some options might include: (1) given the mental model, consider not implementing and waiting for a better time or set of circumstances, (2) just share and explain the mental model and let the leaders decide if they wish to move forward or not, (3) try to meet people “where they are” with their current mental models and collaboratively choose an intervention or implementation strategy consistent with prevailing mental models, or (4) consider methods for how to change the mental models of the intended group(s) through a selected intervention or implementation strategy.

An understanding of mental models may help in the selection of potential implementation strategies. The Expert Recommendations for Implementing Change compilation (ERIC) provides a start with a compilation that lists 73 different implementation strategies in nine different categories (53, 54). However, further research is needed to understand the mechanisms of action for the implementation strategies and how they are activated within different contextual circumstances. By context, we don't just mean the setting, but the individuals, the specific interventions, the infrastructures and processes apparent within the settings, and the interaction of all these factors, as noted in the PRISM diagram (10, 55). Contexts exert strong effects on how and when a strategy may or may not work. We present Table 2 to encourage a conversation about a few example implementation strategies, such as audit and feedback, training and collaborative, and their potential for mental model exploration. We do not propose that it is as simple as identifying mental model issues and matching them with the “right” implementation strategy without considering what the implementation strategy is actually achieving. However, we again offer Figure 1 as possible way to consider mental model challenges and how researchers and quality improvement leaders might navigate through the decision process beginning with identifying if they have a mental model issue and if there is something that can be done about it. Researchers and implementers may also be helped by the conceptualization of how to select implementation strategies by considering both the form (way delivered) and the function (issue to be addressed) as suggested by Perez Jolles et al. (21). Mental models might be one type of issue to be addressed (a function) and the form to be determined by the stakeholders within the context. Additionally, an understanding of mental models is important when considering adaptations. For example, mental models can influence the type and purpose of adaptations made. Figure 3 does not completely address the issue of adaptation explicitly; however, we acknowledge that mental model issues often are part of adaptations as learning occurs through development (56).

**Table 2:** Example implementation strategies with mental model illustrations and examples with the concept of shared decision making.

**Implementation strategy**	**Mental model issue**	**Example with shared decision making**
Audit and feedback	Participants may have a misperception of how they perform relative to others.	Providing audit and feedback may demonstrate the clinicians who believe they are completing SDM at a high level are actually below average.
Training	Inadequate training may leave staff members with a sparse mental model of SDM, lacking important concepts, and causal links.	Staff may not take the time to set up the visit for SDM because their mental model does not predict that it will be important to or improve value for the patients.
Collaborative	Teams may hold different mental models, emphasizing different concepts as key, and featuring different causal beliefs about what actions will affect a quality improvement problem.	Bringing teams together to “get on the same page”: formally elicit mental models and create common understanding around why SDM is important and how it can be organized.

## Considerations for Use of Mental Models in Implementation Research

The concept of mental models is perhaps most useful as a way of seeing the world, such that an explicit understanding of its existence and potential influence may shape the way implementation and research about implementation is approached. Therefore, we hope that one of the primary contributions of this paper is to create a shared mental model about mental models for researchers. It enhances our world view of what is possible in research and what we can do in this field. Although this paper is grounded in implementation research, the closely developing area of improvement science that expands upon quality improvement work may also prove fruitful for those engaged in that work. There may be tools that could be developed for rapid elicitation of mental models for front-line clinicians and quality improvement specialists and their teams.

Given the views presented, it is worth asking–is it possible to change mental models? Yes, of course. This is the purpose of many of the implementation strategies available currently, such as education and training, facilitation, reflection, and audit and feedback. Each has an element of helping individuals and teams consider their beliefs and then have those beliefs questioned, such that each individual has the opportunity to form new belief systems. Interventions do have the ability to make changes, and result in better care (57). Do people actually change their mental models? Sometimes, no, they do not. Deeply held beliefs are often intractable. Yet, knowing about these entrenched beliefs or ways of seeing the world will help us to know how perhaps we can provide information that will facilitate change even in the face of opposing mental models.

The usefulness of mental models in developing effective risk communication has been long established (25). The literature on health care interventions and their effect on mental models as a specific concept is emerging. A common area of research in mental models in health care is the extent to which mental models are shared among a team and the implications of the lack of such shared understanding. In a systematic review, McComb and Simpson (58) found that “Although teamwork and collaboration are discussed frequently in healthcare literature, the concept of shared mental models in that context is not as commonly found but is increasing in appearance.” They note the importance of shared mental models but state the need for further research concerning the impact of shared mental models on healthcare performance to support effective teamwork and collaboration. Some studies, particularly in the fields of emergency medicine and surgery, demonstrate that when mental models differ across team members, it can create problems in implementation (59–63). Conversely, when shared mental models exit, this seems to facilitate implementation (64–66). For example, in a study of veterans facilities, “findings indicated that high-performing facilities exhibited both (a) a clear, focused shared mental model of guidelines and (b) a tendency to use performance feedback as a learning opportunity.” This seems to indicate that shared mental models may be a necessary but not sufficient ingredient for implementation success. While further study is needed to better understand this interplay of implementation strategies and outcomes, some training programs are beginning to encourage teams to explicitly develop shared mental models as a means of improved implementation (67, 68). Additional sentinel information on team mental models, their application and analysis are also available (69–72).

## Discussion

We propose that the consideration of mental models may be a unique lens through which to view implementation research and may benefit the field if further explored and utilized. Mental models strongly influence how individuals and groups think both explicitly and implicitly about tasks and priorities, shape how new initiatives get formed and take off (or not), and have implications for future work in the field. At present it is speculative to describe exactly how mental models can be used to guide implementation and adaptations, but they seem very relevant to help diagnose and address key processes in the steps to successful/failed implementation. Future research is needed to empirically test (a) the predictive validity of mental models compared to other conceptual approaches; and (b) the comparative effectiveness of interventions that include vs. do not include mental models approaches to implementation.

Using a mental models lens to examine implementation work may create new opportunities for intervention and/or implementation strategies to be addressed in new ways or to identify pathways to failed implementation that can provide better understanding of how and why those interventions and/or implementation strategies are not working. We are limited by our current tools, but future research could develop methods by which mental models may be reliably, and pragmatically assessed and used to guide implementation strategies and adaptations.

## Author Contributions

All authors contributed to this paper by writing at least one section. All authors reviewed and approved the final manuscript.

## Conflict of Interest

The authors declare that the research was conducted in the absence of any commercial or financial relationships that could be construed as a potential conflict of interest.
